# Nutrient Sensing by Hypothalamic Tanycytes

**DOI:** 10.3389/fendo.2019.00244

**Published:** 2019-04-16

**Authors:** Roberto Javier Elizondo-Vega, Antonia Recabal, Karina Oyarce

**Affiliations:** ^1^Laboratorio de Biología Celular, Departamento de Biología Celular, Facultad de Ciencias Biológicas, Universidad de Concepción, Concepción, Chile; ^2^Facultad de Medicina y Ciencia, Universidad San Sebastián, Concepción, Chile

**Keywords:** tanycytes, glucosensing, amino acid detection, fatty acids, vitamins, hypothalamus

## Abstract

Nutritional signals have long been implicated in the control of cellular processes that take place in the hypothalamus. This includes food intake regulation and energy balance, inflammation, and most recently, neurogenesis. One of the main glial cells residing in the hypothalamus are tanycytes, radial glial-like cells, whose bodies are located in the lining of the third ventricle, with processes extending to the parenchyma and reaching neuronal nuclei. Their unique anatomical location makes them directly exposed to nutrients in the cerebrospinal fluid. Several research groups have shown that tanycytes can respond to nutritional signals by different mechanisms, such as calcium signaling, metabolic shift, and changes in proliferation/differentiation potential. Despite cumulative evidence showing tanycytes have the molecular components to participate in nutrient detection and response, there are no enough functional studies connecting tanycyte nutrient sensing with hypothalamic functions, nor that highlight the relevance of this process in physiological and pathological context. This review will summarize recent evidence that supports a nutrient sensor role for tanycytes in the hypothalamus, highlighting the need for more detailed analysis on the actual implications of tanycyte-nutrient sensing and how this process can be modulated, which might allow the discovery of new metabolic and signaling pathways as therapeutic targets, for the treatment of hypothalamic related diseases.

## Introduction

**Figure 1:**
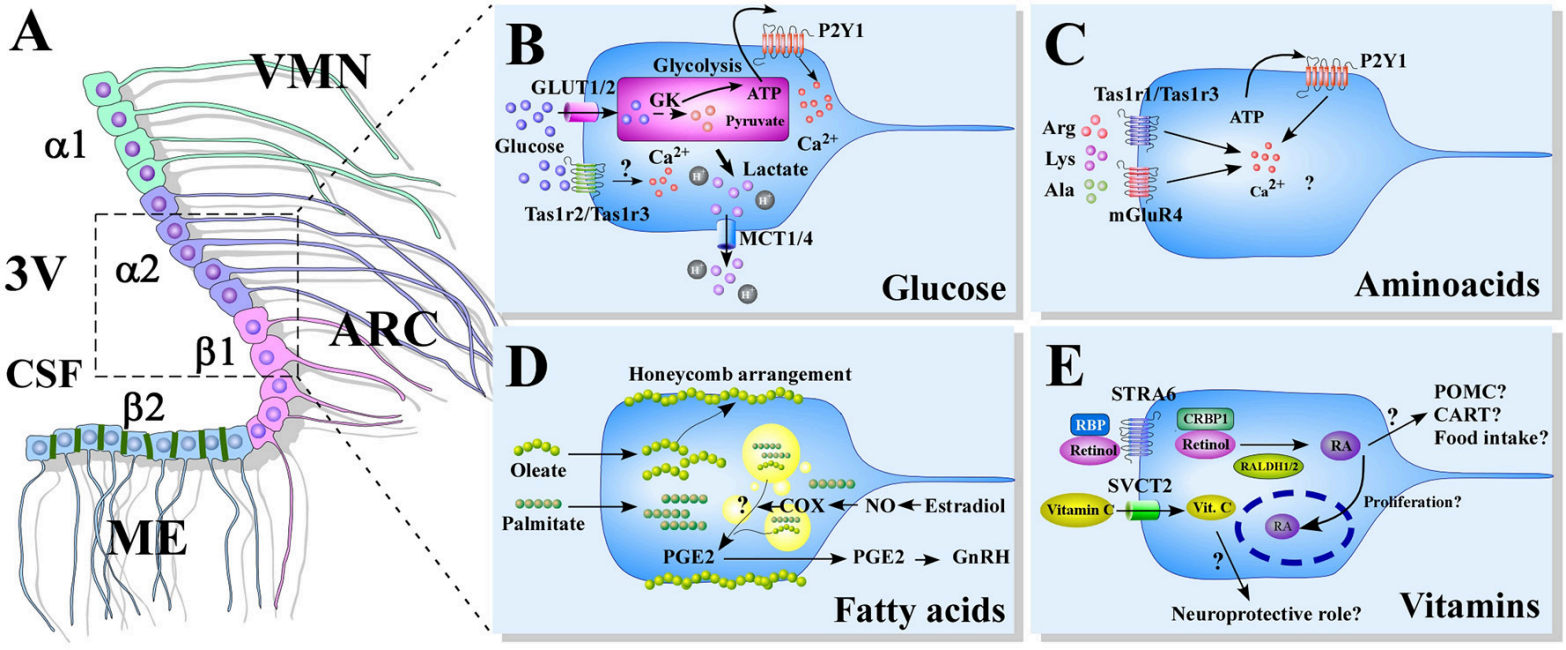
Models of nutrient sensing mediated by tanycytes. **(A)** A schematic representation of the distribution of tanycytes over the wall of the third ventricle (3V). The α1-tanycytes in light green (α1) have long projections that make contact with the neurons of the VMN. α2-tancycytes in purple (α2), have projections to the ARC. β1-tanycytes in pink (β1), make projections to the ARC. Finally, in the floor of the 3V, β2-tanycytes in light blue (β2), are joined by tight junctions forming part of the median eminence (ME)-cerebrospinal fluid (CSF) barrier, and their projections make contact with the ME. **(B–E)** Scheme based on proposed glucose **(B)**, aminoacids **(C)**, fatty acids **(D)**, and vitamins **(E)** sensing mechanism mediated by tanycytes. 3V: third ventricle; CSF, cerebral spinal fluid; ME, median eminenece; ARC, arcuate nucleus; VMN, ventromedial nucleus; GK, glucokinase; PGE2, prostaglandin E2; GnRH, Gonadotropin-release hormone; RBP, Retinol binding protein; STRA6, RBP receptor; CRBP1, cellular retinol-binding protein; RALDH, retinaldehyde dehydrogenase; RA, retinoic acid; POMC, pro-opiomelanocortin; CART, cocaine- and amphetamine-regulated transcript; SVCT2, sodium vitamin C co-transporter 2.

Tanycytes are hypothalamic radial glial-like cells, which are characterized by having their cell bodies located on the basal walls of the third ventricle (3V), in direct contact with the cerebral-spinal fluid (CSF). Histological analysis of rat and mice hypothalamic sections show that tanycytes exhibit long processes that touch blood vessels and neuronal centers located within the ventromedial (VMN) and arcuate nucleus (ARC) (1–3). Tanycytes are classified based on their distribution in the hypothalamic ventricular wall into α1, α2, β1, and β2 (4), (Figure 1A). Basal processes of α1-tanycytes project toward the VMN, while those of α2-tanycytes project to the ARC (1). β1-tanycytes line the infundibular recess, and their basal projections reach the lateral regions of the median eminence (ME) and the ARC; while β2-tanycytes cover the floor of the 3V and extend their projections inside the ME to contact fenestrated capillaries (5–7). This provides privileged access to nutritional signals carried by the bloodstream (8, 9). Also, β2-tanycytes are connected to each other through tight junctions (10), forming a physical barrier for polar molecules that prevent their diffusion into the brain via the CSF, known as the ME-CSF barrier (5, 11, 12).

Importantly, accumulating recent data showing increased heterogeneity of tanycytes populations, in terms of neurogenic potential, marker expression, structural differences and transcriptomic profile, poses the need of reviewing the current classification to find new ways of better describing the function of tanycytes subpopulations, so new classification systems have emerged (13).

These anatomical features make it feasible to propose that tanycytes have the properties to detect and respond to different nutritional signals, while being able to functionally couple with neurons of the hypothalamic region. In this review, we will summarize the existing data regarding tanycytes involvement in different nutrients sensing, such as glucose, lipids, vitamins, and amino acids.

## Glucosensing Mediated by Tanycytes

Plasma glucose concentrations are known to modulate appetite, and therefore regulate food intake initiation and termination. This is done in a process that is centrally regulated by the hypothalamus, where rises in plasma glucose concentration that occur after a meal signal meal termination (14), although the mechanisms by which it enters the brain are not fully understood. Through microdialysis studies in rats, it has been demonstrated that glucose concentration in the parenchyma does not vary significantly (15), while glucose concentration in CSF changes proportionally to variations in blood glucose concentration (16, 17), suggesting the existence of a mechanism that facilitates the efficient transfer of glucose from the blood to CSF (18). Glucose uptake into the CSF would occur through the circumventricular organs, which are structures that are part of the ventricular walls and have fenestrated capillaries, which gives them high permeability to different nutrients (19).

In the hypothalamus, the ME is a circumventricular organ and, as is was noted before, the ME-CSF barrier is composed of β2-tanycytes, therefore tanycytes would be the main regulators of nutrients accessibility to the CSF (18).

In this context, studies on monkey and rodent hypothalamic slices show that α and β1-tanycytes, express high levels of glucose facilitative transporter GLUT1, exhibiting an intense immunoreaction in their cell processes that reach hypothalamic capillaries and the ARC (1, 20). In addition, β1 and β2-tanycytes express GLUT2, a low affinity/high-capacity glucose transporter. The expression of these two glucose transporters allows tanycytes to incorporate glucose from the CSF (21).

Moreover, rat tanycytes express glucokinase (GK) *in situ* and *in vitro* (22), an enzyme that at high glucose concentrations has an efficient glucose phosphorylation activity, and also express the regulatory glucokinase protein (GKRP) (23), which regulates the activity and nuclear compartmentalization of GK (24, 25). The expression of GLUT2, GK, and GKRP in tanycytes puts them in a privileged position to sense glucose variations in the CSF (3).

It has been established that rat primary cultures of tanycytes in response to an increase in extracellular glucose levels, augment intracellular Ca^2+^ concentration ([Ca^2+^]i) and ATP levels, a response that is dependent on glycolytic metabolism and P2Y1 receptor activation (26). *In situ* analysis of hypothalamic slices acutely exposed to glucose or non-metabolizable analogs on the tanycytes bodies also show [Ca^2+^]i increase in an ATP-dependent manner (27–29) (Figure 1B).

The physiological relevance of glucose or glucose analogs-induced responses on tanycytes has not been fully studied. However, some groups have made progress on demonstrating the importance of glucose metabolism in tanycytes and how this can impact the activity of hypothalamic neuronal nuclei. For instance, *in vitro* and *in situ* studies in rodents, showing monocarboxylate transporter (MCT) expression in tanycytes have led to propose that glucose would be preferentially captured by tanycytes, generating other metabolic substrates, like lactate (3, 30, 31), that once released, it could be used by nearby neurons in a mechanism similar to one originally described by Pellerin and Magistretti, known as the astrocyte-neuron lactate shuttle hypothesis (32). *In vivo* studies in rats, inhibiting monocarboxylate transporter 1 (MCT1), GLUT2 or GK in tanycytes, through adenoviral injections into the 3V, show impaired neuronal response to fasting and acute glucose administration, given by altered expression of orexigenic and anorexigenic neuropeptides, accompanied by altered feeding behavior (33–35). These recent data support the notion that metabolic coupling between tanycytes and neurons can modulate the neuronal activity in hypothalamic areas associated with regulation of eating behavior.

Interestingly, a study in male siberian hamster showed, through *in situ* hybridization, that MCTs could be subject to photoperiodic regulation (36), suggesting that the amount of lactate released by tanycytes could be affected by daylight duration. In addition, the authors propose that lactate can be used for the synthesis of glutamine, modulating the supply of glutamate neurotransmitter to hypothalamic neurons (36).

It is important to note that a recent study has shown that tanycytes are able to sense glucose by a different mechanism, through the sweet taste receptor Tas1r2/Tas1r3, whose activation with glucose and other analogs produces [Ca^2+^]i increase (28). It is necessary to further explore if this mechanism is truly relevant under physiological conditions.

## Amino Acid Detection by Tanycytes

Different studies have analyzed the impact of protein-rich or protein-deprived diets on brain functions, such as central regulation of energy balance and food intake showing that protein consumption is homeostatically regulated by central amino acid sensing (37, 38). One of the sensing centers is localized in the hypothalamus. This is shown in studies where hypothalamic microinjections of either balanced amino-acid solutions or just leucine suppresses appetite and inhibits feeding in rats (39–43). Other studies with leucine supplementation in the diet also show feeding inhibition, similar to that observed with high-protein diets (44, 45). In addition, plasma leucine levels increase rapidly after food intake (46), also increasing its concentration in the brain (47), exercising a suppressive role on food intake.

Regarding amino acid entry into the brain, it has been determined that endothelial cells from the BBB express diverse systems of amino acid transporters *in situ*. So far, four facilitative carriers have been identified in the luminal (blood) membrane; system “L1” (for large essential neutral amino acid), “y^+^” (for cationic amino acids), “xG^−^” (for acidic amino acid) and “n” (for glutamine). The abluminal (parenchyma) membrane, on the other hand, only expresses system L1 and y+, (with lower and higher expression compared to the luminal membrane, respectively) (48–50). This differential distribution allows for the concentration of essential neutral amino acids, while promoting the elimination of non-essential amino acids and toxic amino acids such as glutamate.

The abluminal membrane, in addition, expresses 5 systems of Na^+^ -dependent amino acid transporters that allow their export from the brain (51–53). System A (for small nonessential neutral amino acids, that preferentially transport alanine) (54–56); ASC system (for some large and small neutral amino acids) (57–59), system N (for nitrogen rich amino acids) (60), the excitatory acidic amino acid transporter (EAAT) family (61, 62), and finally the Na^+^ -LNAA system (for large neutral amino acids) (50).

Most of the literature describes hypothalamic amino acid sensing relying on some discrete groups of neurons, located at the basal and lateral hypothalamus. For instance, it has been shown, in rodents, that leucine increases the frequency of POMC action potentials *ex vivo* and c-Fos immunoreactivity *in situ*, in a mTOR dependent manner (40, 42, 63). In addition, leucine effects on food intake are neutralized with mTORC1 inhibitor rapamycin (63). Other reports using mice show that hypocretin/orexin neurons also respond to non-essential amino acids through a dual mechanism involving inhibition of K^+^(ATP) channels and activation of amino acid transporters from system A *ex vivo* (64).

New evidence, however, suggests that tanycytes might also contribute to hypothalamic amino acid sensing, highlighting the involvement of non-neuronal cells in this process. Benford et al. (28) showed in rodents that glucosensing mediated by a group of tanycytes, was dependent on the expression of the sweet taste receptor Tas1r2/Tas1r3 (28), which shares structural similarities with the Tas1r1/Tas1r3 heterodimer associated with the umami taste or non-aromatic L-amino acids detection (65, 66). 67, on the other hand, showed using rodent brain slices that L-amino acids such as Arg, Lys, and Ala increased [Ca^2+^]i in a ATP-dependent manner in tanycytes, a response also dependent on Tas1r1 expression and mGluR4 function, which is another umami taste receptor (67).

These new findings show that tanycytes have at least two mechanisms of amino acid sensing through umami taste receptors, proposing tanycytes as a new cell type for hypothalamic amino acid detection (Figure 1C). More studies are needed to address the relevance and implications of amino acid glial detection on regulating food intake, as well as whether these mechanisms might be altered in pathologies associated with diabetes and obesity.

## Fatty Acid Sensing Mediated by Tanycytes

Although several studies have emerged showing high fat diet (HFD) or specific fatty acid administration can induce hypothalamic inflammation (68–70) and/or hypothalamic neurogenesis (71, 72), not many studies have addressed the particular involvement of tanycytes in fatty acid incorporation and response.

It has been shown that the BBB limits the free passage of lipid components into the brain in a selective way (73, 74), allowing for example, the entry of linoleic acid to the brain parenchyma, while restricting the entry of oleic acid (74). Recent work by Hofmann et al. (75), using click chemistry to trace fatty acids in the hypothalamus, shows a differential distribution when slices are acutely incubated with saturated (SFA) vs. poly-unsaturated fatty acids (PUFAs). For instance, SFAs palmitate and stearate were highly detected in tanycytes and in a lesser degree in the ARC, while PUFAs oleate and linoleate were found in both areas, with signal diffusing from the ventricular wall to the parenchyma (75). Oleate staining in tanycytes was mostly isolated to the plasma membrane or spread in between tanycytes, resulting in a honeycomb-like arrangement, while palmitate was highly detected in the whole tanycytic layer (75).

This hypothalamic fatty acid distribution seems to change when mice are kept on a HFD for 10 weeks, observing a reduction of oleic acid presence in the ARC with a more concentrated expression in the tanycyte layer. In these same mice, HFD increased the number and size of lipid droplets (75), indicating tanycytes capability to store and perhaps metabolize these lipids. In a recent review, Rodriguez et al., suggest that lipid droplets present in tanycytes could be used in the production of prostaglandin E2 (PGE2) (76), which has been describe to promotes the retraction of tanycytes endfeet, facilitating the secretion of gonadotrophin releasing hormone (GnRH) into the pericapillary space and fenestrated capillaries, reviewed in (77) (Figure 1D).

However, how these observations relate to tanycytes role in hypothalamic energy balance control, inflammation or neurogenesis are still lacking. Because fatty acids can act as signaling molecules and regulators of the above processes, more studies are needed to elucidate if they can alter the way tanycytes sense other molecules or change their responses.

## Tanycytes and Vitamins

Although several specific roles related to brain function have been attributed to different vitamins (78), only vitamin A has been largely studied in the hypothalamus. More specifically in describing their key role relating to proteins for uptake and metabolism. However, not much is known about vitamin A possible effects on hypothalamic regulated functions.

This liposoluble vitamin, known as retinol, travels through the bloodstream bound to the retinol binding protein (RBP) and can enter cells through the interaction with RBP receptor (STRA6) (79). Once inside the cell, it interacts with cellular retinol-binding protein (CRBP1), allowing the proximity to enzymes, called retinaldehyde dehydrogenases (RALDHs), that convert retinol into its metabolic active form, retinoic acid (RA) (80, 81). RA later interacts with nuclear receptors (RARs), which act as ligand-activated transcription factors that when bound to retinoid X receptors (RXRs), translocate to the nucleus, interact with RA response elements (RARE) and regulate gene expression (82).

Vitamin A immunohistochemistry on rat brain tissue, with an antiserum developed against RA shows that vitamin A is almost exclusively detected in hypothalamic cell bodies and some dendrites of the paraventricular nuclei and the dorsal perifornical region (83). Interestingly, studies have shown that some of the crucial proteins involved in vitamin A uptake and nuclear transportation, as well as RA synthetizing enzymes are expressed by tanycytes and that expression can be regulated by melatonin and thyroid hormones (84–86). (84), showed in adult rats that tanycytes were the only cells in the hypothalamus positive for RALDH1 expression, by immunofluorescence (84, 86). Moreover, RALDH2 was shown to be expressed in tanycytes at the protein level, but not mRNA *in situ*, and because RALDH2 proteins were detected by western blotting also in the choroid plexus and CSF, the authors suggested that tanycytes might be able to endocyte it from the 3V (86). They also showed the expression of STRA6 and CRBP1 in tanycytes by *in situ* hybridization (84) (Figure 1E).

In rodents, numerous studies have seen that RA related proteins are under photoperiodic control in tanycytes. For instance, in the siberian hamster, exposure to long photoperiod (LD) correlates to increase levels of CRBP1 *in situ*, particularly in the lining of the 3V, while short photoperiod (SD) correlates with very low levels of CRBP1 (87). In addition, pinealectomized animals maintained higher levels of CRBP1, mimicking LD, showing at least in some way CRBP1 expression was modulated by melatonin, which has also been shown to regulate the expression of other proteins on tanycytes, such as cytoskeleton intermediate filament protein vimentin (88).

In the photosensitive rat strain F344/N, LD induces high expression of STRA6, CRBP1, RALDH1, and CYP26b1, a monooxygenase involved in RA metabolism, in the lining of the 3V, while low or undetectable expression is detected under SD *in situ* (85). This photoperiod regulation of RA related genes can also be achieved by melatonin injections under LD, recapitulating the results obtained with pinealectomized mice by Ross et al. (87).

Another study, using 8 week/old rats, RALDH1 levels in the hypothalamus increase at the transcriptional level after 4 h of receiving a triiodothyronine (T3) subcutaneous injection. They also increase when hypothalamic slices are directly incubated for 48 h with T3 *ex vivo* or when primary cultures of tanycytes are treated with T3 *in vitro* (89). In addition, CYP26b1, known to be upregulated by RA, increases its expression by 2-fold in the hypothalamic area when T3 is administered *in vivo*, suggesting RA synthesis occurred on tanycytes (89).

All of this data shows tanycytes are able to synthesize and metabolize RA, in a process regulated by both melatonin and T3, however, only few studies correlate RA proteins expression patterns to cellular processes that could be in part regulated by tanycytes. For example, in hypothalamic organotypic slice cultures, the addition of RA blocks tanycytes proliferation induced by epidermal growth factor. While *in vivo*, RALDH1 expression in tanycytes during LD correlates with significantly lower number of cells positive for proliferation marker KI67 (86). No further studies have seen if this anti-proliferative effect modulates hypothalamic neurogenesis, as it has been shown for RA in other neurogenic niches as the subventricular zone and the dentate gyrus (90–92).

Finally, rat hypothalamic slices cultured with 1 μM RA increased the transcriptional expression of AgRP and POMC neuropeptides, but the same experiment in the photosensitive rat strain F344/N showed only significant increase for POMC expression (89). More studies aiming to suppress or overexpress RA related proteins in tanycytes are needed to study whether endogenous RA synthesis and metabolism can modulate energy balance and food intake processes, as well as other cellular processes involving tanycytes participation.

Regarding other vitamins, it might be worth mentioning that sodium- vitamin C co-transporter 2 (SVCT2) has been detected in rat and mice tanycytes, by *in-situ* hybridization, confocal microscopy and ultrastructural immunohistochemistry, showing a specific location in β-1 tanycytes (21, 93). Although vitamin C have long been known to participate in numerous brain functions, such as neurotransmission, neuroprotection and differentiation (78), there are no studies exploring the specific role that vitamin C might have in tanycytes, or if tanycytes only accumulate vitamin C to later release it into the parenchyma as a neuroprotective agent. Taking into consideration that tanycytes are proposed as hypothalamic stem cells, and that vitamin C increases neurogenesis *in vitro* (94, 95) it would be interesting to analyze how vitamin C uptake by tanycytes can modulate this process *in vivo*.

## Future Directions

Due to their localization in the hypothalamus, tanycytes are in a privileged position to detect different signals, such as hormones, nutrients and growth factors, coming either from the CSF or the blood. Nevertheless, demonstrating that tanycytes act as a sensory cell, able to modulate other brain cell types during regulatory complex processes, has taken a long time. In this context, energy homeostasis and neuroendocrine regulation mediated by hormonal responses that increase energy expenditure or change feeding behavior, require fine-tune regulation, in which brain detection of nutrients plays a key role. For a long time, studies that have tried to understand the regulatory mechanisms involved in nutrient detection have focused on the neuronal component, neglecting the participation of a cell type that, molecularly and histologically, has the necessary characteristics to participate in these processes. Here, we present a small compilation of the evidence supporting the micronutrient sensor role of tanycytes, which altogether suggest that it changes the nutrient sensing ability, which could contribute to the physiopathological responses observed in metabolic disease like obesity and diabetes.

However, as it can be extracted from the literature, for some micronutrients, scientific evidence is still scarce, and further studies are needed to delve into the molecular and cellular mechanisms behind tanycytes nutrients sensing, as well as to explore their physiological or pathological relevance. It is necessary to incorporate genetic tools in these studies that allow for specific tracing of the different tanycytes populations, complemented with *in vivo* analysis of metabolic related responses, in order to improve tanycyte sensing related knowledge.

## Author Contributions

RE-V and KO wrote the manuscript with the input of AR.

### Conflict of Interest Statement

The authors declare that the research was conducted in the absence of any commercial or financial relationships that could be construed as a potential conflict of interest.
